# Uptake of Cervical Cancer Screening and Associated Factors among Women in Rural Uganda: A Cross Sectional Study

**DOI:** 10.1371/journal.pone.0149696

**Published:** 2016-02-19

**Authors:** Rawlance Ndejjo, Trasias Mukama, Angele Musabyimana, David Musoke

**Affiliations:** 1 Department of Disease Control and Environmental Health, School of Public Health, College of Health Sciences, Makerere University, Kampala, Uganda; 2 Department of Community Health, School of Public Health, College of Medicine and Health Sciences, University of Rwanda, Kigali, Rwanda; Istituto Nazionale Tumori, ITALY

## Abstract

**Background:**

In developing countries, inadequate access to effective screening for cervical cancer often contributes to the high morbidity and mortality caused by the disease. The largest burden of this falls mostly on underserved populations in rural areas, where health care access is characterized by transport challenges, ill equipped health facilities, and lack of information access. This study assessed uptake of cervical cancer screening and associated factors among women in rural Uganda.

**Methods:**

This descriptive cross sectional study was carried out in Bugiri and Mayuge districts in eastern Uganda and utilised quantitative data collection methods. Data were collected using a semi-structured questionnaire on cervical cancer screening among females aged between 25 and 49 years who had spent six or more months in the area. Data were entered in Epidata 3.02 and analysed in STATA 12.0 statistical software. Univariate, bivariate and multivariate analyses were performed.

**Results:**

Of the 900 women, only 43 (4.8%) had ever been screened for cervical cancer. Among respondents who were screened, 21 (48.8%) did so because they had been requested by a health worker, 17 (39.5%) had certain signs and symptoms they associated with cervical cancer while 16 (37.2%) did it voluntarily to know their status. Barriers to cervical cancer screening were negative individual perceptions 553 (64.5%) and health facility related challenges 142 (16.6%). Other respondents said they were not aware of the screening service 416 (48.5%). The independent predictors of cervical cancer screening were: being recommended by a health worker [AOR = 87.85, p<0.001], knowing where screening services were offered [AOR = 6.24, p = 0.004], and knowing someone who had ever been screened [AOR = 9.48, p = 0.001].

**Conclusion:**

The prevalence of cervical cancer screening is very low in rural Uganda. Interventions to increase uptake of cervical cancer screening should be implemented so as to improve access to the service in rural areas.

## Introduction

Worldwide, over 85% of cervical cancer deaths every year occur in developing countries [1]. This is attributed to inadequate access to effective screening which results in to less recognition of the disease during its early stages and higher chances of it developing to advanced stages with poor prospects of treatment [1]. Indeed, over 80% of cancers in sub-Saharan Africa are detected in their late stages [2, 3]. In contrast, developed countries have programmes to enable effective screening and thus pre-cancerous lesions are identified and treated early enough [1]. Uganda ranks 14^th^ among countries with the highest incidence of cervical cancer, and over 65% of those diagnosed with the disease die from it [4]. It is estimated that about 33.6% of women in the general population in Uganda harbour cervical human papilloma virus infection—the main cause of cervical cancer at any given time [5]. The World Health Organization recommends screening and vaccination programmes throughout the sub-Saharan African region [6]. The government of Uganda thus launched its strategic plan for cervical cancer prevention and control in 2010 with a target of screening and vaccinating 80% of the eligible persons by 2015 [7].

Cervical cancer is potentially preventable and effective screening programmes can lead to reduced morbidity and mortality [3, 8]. The success of screening depends on access and up-take, quality of screening tests, adequacy of follow-up, and diagnosis and treatment of lesions detected. Coverage of cancer screening services is lowest in low and middle income countries averagely estimated at 19%. This is partly due to the presence of only a few trained and skilled health workers, and lack of healthcare resources to sustain screening programmes [9, 10]. Studies have shown that only a small percentage (6%-27%) of women in sub-Saharan Africa report having received cervical cancer screening [11–16]. This is even lower in the East African region where cervical cancer age-standardized incidence rates are highest [17].

The largest burden of cervical cancer mortality falls mostly on underserved populations [10]. Poor, older and rural women who have higher risks of developing cervical cancer are less likely to be screened [10]. Access to health services in rural areas, has been characterized by transport challenges, long distances to health centres, ill equipped health facilities and lack of information access. Studies about cervical cancer screening carried out in the East African region have mainly focused on urban areas and health care settings [11, 14, 15, 18, 19]. These studies have shown that there are various gaps in knowledge on cervical cancer in the studied communities [11, 14–16, 19]. The studies further report other barriers to accessing screening services including cultural constraints/beliefs about the illness, economic factors, domestic gender power relations, alternative authoritative sources of reproductive health knowledge and unfriendly health care services [11, 12, 19]. In addition, illiteracy, belief in not being at risk, having many contending issues, nonchalant attitude to personal health, financial constraints, and fear of having a positive result have been frequently cited [12, 18, 20, 21]. Considering the challenges faced in accessing cervical cancer screening in rural areas, it is important to understand the uptake of these services and factors that affect their utilisation so as to inform effective interventions. This study assessed uptake of cervical cancer screening and associated factors among women in rural Uganda.

## Methods

### Study design and data collection

This was a descriptive cross sectional study that utilised quantitative data collection methods. Using the sample size estimation formula for cross sectional studies [22] with Z = 1.96, p = 0.5 and a precision of 5% taking into account a design effect of 2.0 and a non-response rate of 10%, a minimum sample size of 845 respondents was obtained. Data were collected on cervical cancer and screening among females aged between 25 and 49 years who had spent six or more months in the area using a semi-structured questionnaire. The questionnaire was developed in English and then translated to *Lusoga*, the main local language used in the study area. The pretested questionnaire assessed the respondents’ knowledge about cervical cancer and screening including knowledge of recommended age for screening, importance of early screening, cervical cancer preventive measures, symptoms of cervical cancer, and screening tests for cervical cancer. Additionally, it had questions regarding access to screening services including whether respondents were screened and why, where they accessed the cervical cancer screening service, what motivated them to access it and the number of times they had been screened for cervical cancer. The questionnaire also had questions on the health facility factors including where respondents accessed reproductive health services, their ease of access to reproductive health care, distance to health facilities, whether they had ever been recommended for screening by a health worker and their knowledge of where cervical cancer screening was offered. The other section of the questionnaire collected socio demographic information such as age, education levels, occupation, socio economic status, parity, and marital status. A team of trained research assistants administered the questionnaire to respondents in their homes. Only one participant was selected per sampled household with priority given to household heads or their spouses.

### Study area and sampling

The study was carried out in Bugiri and Mayuge districts in eastern Uganda located approximately 150 kilometres from Kampala, the capital city of Uganda. These districts in 2014 had a population of 870,000 of whom 51.4% were females [23] and a combined area of 10,372 km square. Being predominantly rural districts, most residents engage in subsistence farming and a few operate small businesses in trading centres. The districts are located along the shores of Lake Victoria and communities that border the lake are involved in fishing. Cervical cancer screening services in the two districts are provided by Bugiri district hospital which also serves other neighbouring districts. In addition, two private health facilities, both located in Bugiri town, provide cancer screening services in Bugiri district and one private facility serves Mayuge district. In the study, sampling was carried out at different stages as follows: five sub counties out of nine from Bugiri and seven from Mayuge district were randomly selected. Five villages were then selected from each sub county using random sampling while systematic random sampling was utilised to select households that participated in the study.

### Data analysis

Data were entered in Epidata version 3.02 (EpiData Association, Denmark) and analysed in STATA version 12.0 (Stata Corp, Texas, USA) statistical software. The outcome variable was screening for cervical cancer and was assigned 1 when a respondent reported to have ever been screened and 0 when otherwise. The exploratory variables consisted of respondents’ socio-demographic factors, health facility factors, and knowledge regarding cervical cancer and screening. Bivariate analysis was carried out to determine the relationship between each of the different exploratory variables and the outcome variable. Basing on biological plausibility and p<0.2, variables from the bivariate analyses were added to a multivariate logistic regression model and a backward stepwise logistic regression performed. Odds ratios and p-values were used as measures of association with a p-value of less than 0.05 being considered for a statistically significant relationship at the 95% confidence level.

### Ethical considerations

This study was approved by the Makerere University School of Public Health Higher Degrees, Research and Ethics Committee and registered by the Uganda National Council for Science and Technology. Participation in the study was voluntary and participants provided written informed consent only after being explained to the study aims, benefits and potential risks.

## Results

### Socio demographic characteristics of respondents

Among the 900 respondents, majority 703 (78.1%) belonged to the 25–39 years age group, 767 (85.2%) were married and 622 (69.1%) earned less than 40 US dollars per month. Only a quarter of respondents 228 (25.3%) attained post primary education, slightly more than half 502 (55.8%) were farmers while two thirds 610 (67.8%) resided in rural areas (Table 1).

**Table 1:** Socio demographic characteristics of respondents (N = 900).

Characteristic	Categories	Frequency (%)
District	Bugiri	452 (50.2)
	Mayuge	448 (49.8)
Residence	Rural	610 (67.8)
	Semi-urban/urban	290 (32.2)
Age (years)	Mean (SD)	32.9 (6.7)
	25–39	703 (78.1)
	40–49	197 (21.9)
Education level	None/primary	672 (74.7)
	Post primary	228 (25.3)
Religion	Muslims	382 (42.4)
	Christians	518 (57.6)
Marital status	Single	133 (14.8)
	Married	767 (85.2)
Nature of marriage (n = 767)	Monogamous	465 (60.6)
	Polygamous	302 (39.4)
Occupation	Farming	502 (55.8)
	Others (business, housewife, civil servant)	195 (21.7)
Parity	Mean (SD)	5.04 (2.7)
	Four and below	430 (47.8)
	Above four	470 (52.2)
Average monthly household income	Less than $40	622 (69.1)
	$40 and above	278 (30.9)
Household head	Yes	143 (15.9)
	No	757 (84.1)
Ever tested for HIV	Yes	756 (84.0)
	No	144 (16.0)
Reported HIV status (n = 730)	Negative	708 (96.1)
	Positive	22 (3.0)
Ever used modern family planning method	Yes	583 (64.8)
	No	317 (35.2)
Number of persons in household	Five and below	358 (39.8)
	Above five	542 (60.2)

### Uptake of cervical cancer screening

Among the respondents, 43 (4.8%) had ever been screened for cervical cancer. Most of these 35 (81.4%) accessed the service from a government health facility with the rest getting it from private facilities. Most respondents 25 (58.1%) had undergone the procedure within the preceding 12 months and only 14 (32.5%) had ever been screened two or more times. Respondents who had been screened did so because they had been requested by health workers 21 (48.8%), had certain signs and symptoms 17 (39.5%) while 16 (37.2%) did it voluntarily to know their status. Among respondents who had not been screened, most 553 (64.5%) stated personal perception related reasons (having no signs and symptoms of the disease, not being at risk, lack of time and fear of test outcomes). Others said they were not aware of cervical cancer screening services 416 (48.5%) while the rest 142 (16.6%) stated health facility related challenges (distance, costs and long waiting times at facilities).

### Socio-demographic factors associated with uptake of cervical cancer screening

Bugiri district had more respondents who had been screened for cervical cancer 27 (6.0%) than Mayuge district 16 (3.6%). Living in semi urban or urban areas was significantly associated with having undergone cervical cancer screening [COR = 2.54 (95% CI: 1.37–4.71), p = 0.003]. Respondents who lived in households with five or less members were twice more likely to have undergone cervical cancer screening than their counterparts [COR = 2.18 (95% CI: 1.17–4.07), p = 0.014]. Those who had ever tested for HIV were four times more likely to have undergone cervical cancer screening compared to those who had never done the test although this was not statistically significant [COR = 4.07 (95% CI: 0.97–17.02), p = 0.054] (Table 2).

**Table 2:** Association between socio-demographic characteristics and uptake of cervical cancer screening.

Characteristic	Screened (%)	COR (95% CI)	p-value
**District**			
Bugiri	27 (6.0)	1	
Mayuge	16 (3.6)	0.58 (0.31–1.09)	0.095
**Residence**			
Rural	20 (3.3)	1	
Semi-urban / urban	23 (7.9)	2.54 (1.37–4.71)	0.003*
**Age (years)**			
25–39	31 (4.4)	1	
40–49	12 (6.1)	1.41 (0.71–2.79)	0.330
**Education level**			
None/primary	32 (4.8)	1	
Post primary	11 (4.8)	1.01 (0.50–2.04)	0.969
**Religion**			
Muslims	17 (4.4)	1	
Christians	26 (5.0)	1.13 (0.61–2.12)	0.693
**Marital status**			
Single	6 (4.5)	1	
Married	37 (4.8)	1.07 (0.44–2.59)	0.876
**Nature of marriage**			
Polygamous	19 (6.3)	1	
Monogamous	18 (3.9)	0.60 (0.31–1.16)	0.130
**Occupation**			
Farming	19 (3.8)	1	
Other (business/trade, housewife, civil servant)	24 (6.0)	1.63 (0.88–3.02)	0.120
**Parity**			
Above four	20 (4.3)	1	
Four and below	23 (5.3)	1.27 (0.69–2.35)	0.443
**Household income**			
Less than $40	28 (4.5)	1	
Above $40	15 (5.4)	1.21 (0.64–2.30)	0.562
**Household head**			
No	34 (4.5)	1	
Yes	9 (6.3)	1.43 (0.70–3.05)	0.356
**Ever tested for HIV**			
No	2 (1.4)	1	
Yes	41 (5.4)	4.07 (0.97–17.02)	0.054
**Reported HIV status**			
Negative	39 (5.5)	1	
Positive	2 (9.1)	1.71 (0.39–7.60)	0.477
**Ever used modern family planning method**			
No	15 (4.7)	1	
Yes	28 (4.8)	1.01 (0.53–1.93)	0.962
**Number of persons in household**			
Above five	18 (3.3)	1	
Five and below	25 (7.0)	2.18 (1.17–4.07)	0.014*

* Statistically significant at p < 0.05.

### Knowledge factors associated with uptake of cervical cancer screening

Among the respondents, knowing at least one test method for cervical cancer was positively associated with having screened for the disease [COR = 2.88 (95% CI: 1.48–5.60), p = 0.002]. Respondents who knew someone who had ever been screened [COR = 8.21 (95% CI: 3.88–17.36), p<0.001) or diagnosed [COR = 2.34 (95% CI: 1.27–4.34), p = 0.007] with the disease were eight and two times more likely to have been screened respectively compared with their counterparts (Table 3).

**Table 3:** Association between knowledge factors and uptake of cervical cancer screening.

Characteristic	Screened (%)	COR (95% CI)	p-value
**Early detection of cervical cancer is helpful**			
No	4 (8.7)	1	
Yes	39 (4.6)	0.50 (0.17–1.47)	0.209
**Cervical cancer is curable if detected early**			
No	8 (3.5)	1	
Yes	35 (5.2)	1.52 (0.69–3.32)	0.295
**Cervical cancer can be prevented**			
No	8 (2.9)	1	
Yes	35 (5.6)	1.98 (0.91–4.32)	0.087
**I am at risk of getting cervical cancer**			
No	8 (3.7))	1	
Yes	35 (5.1)	1.40 (0.64–3.07)	0.398
**Knew recommended age for start of screening**			
No	40 (4.6)	1	
Yes	3 (8.1)	1.81 (0.53–6.16)	0.339
**Knew that one can be vaccinated against cervical cancer**			
No	11 (3.4)	1	
Yes	32 (5.5)	1.66 (0.82–3.33)	0.157
**Knew more than one preventive measure for cervical cancer**			
No	26 (4.1)	1	
Yes	17 (6.3)	1.56 (0.83–2.93)	0.165
**Knew more than one symptom of cervical cancer**			
No	15 (3.6)	1	
Yes	28 (5.7)	1.61 (0.85–3.06)	0.145
**Knew atleast one test for cervical cancer**			
No	13 (2.7)	1	
Yes	30 (7.3)	2.88 (1.48–5.60)	0.002*
**Knew someone who had ever been screened for cervical cancer**			
No	9 (1.5)	1	
Yes	34 (11.2)	8.21 (3.88–17.36)	<0.001*
**Knew someone who had ever been diagnosed with cervical cancer**			
No	22 (3.5)	1	
Yes	21 (7.8)	2.34 (1.27–4.34)	0.007*
**Know someone who had ever died from cervical cancer**			
No	25 (4.0)	1	
Yes	18 (6.7)	1.75 (0.94–3.26)	0.079

* Statistically significant at p < 0.05.

### Health facility factors associated with uptake of cervical cancer screening

The facility where women accessed reproductive health care [COR = 9.71 (95% CI: 1.33–71.11), p = 0.025], their ease of access to this care [COR = 2.27 (95% CI: 1.15–4.48), p = 0.018], having ever been recommended for screening by a health worker [COR = 77.13 (95% CI: 33.85–175.74), p <0.001] and their knowledge of a place where screening was offered [COR = 11.90 (95% CI: 4.64–30.54), p<0.001] were significantly associated with having undergone cervical cancer screening (Table 4).

**Table 4:** Association between health facility factors and uptake of cervical cancer screening.

Characteristic	Screened (%)	COR (95% CI)	p-value
**Place where health care is sought when sick**			
Private facility	6 (3.4)	1	
Government facility	37 (5.1)	1.52 (0.63–3.67)	0.346
**Place where reproductive health care is accessed**			
Private facility	1 (0.6)	1	
Government facility	42 (5.7)	9.71 (1.33–71.11)	0.025*
**Ease of getting reproductive health care**			
Very/somewhat difficult	12 (2.9)	1	
Not difficult	31 (6.4)	2.27 (1.15–4.48)	0.018*
**Biggest problem in accessing reproductive health care**			
Other problem	23 (4.3)	1	
No problem	20 (5.5)	1.30 (0.70–2.41)	0.399
**Ever been recommended for screening by health worker**			
No	8 (1.0)	1	
Yes	35 (43.2)	77.13 (33.85–175.74)	<0.001*
**Knew where cervical cancer screening was provided**			
No	5 (0.9)	1	
Yes	38 (10.2)	11.90 (4.64–30.54)	<0.001*
**Distance to health facility where screening was done (n = 372)**			
5km or more	15 (8.9)	1	
Less than 5km	23 (11.3)	1.29 (0.65–2.57)	0.458
**Distance to nearest health facility**			
5km or more	0 (0.0)		
Less than 5km	43 (5.7)	-	-

* Statistically significant at p < 0.05.

### Independent predictors of uptake of cervical cancer screening

Table 5 shows the independent predictors of uptake of cervical cancer screening among women in rural Uganda. When potential confounders were controlled for, respondents who had been recommended for screening by a health worker were 87 times more likely to have been screened for cervical cancer [AOR = 87.85 (95% CI: 30.28–254.84), p<0.001]. Those who knew where cervical cancer screening services were provided were 6 times more likely to have undergone the procedure [AOR = 6.24 (95% CI: 1.81–21.56), p = 0.004] while those who knew someone who had ever been screened where 9 times more likely to have screened for the disease [AOR = 9.48 (95% CI: 2.39–37.56), p = 0.001] (Table 5).

**Table 5:** Independent predictors of uptake of cervical cancer screening.

Characteristic	AOR (95% CI)	p-value
**District**		
Bugiri	1	
Mayuge	0.62 (0.19–1.96)	0.417
**Residence**		
Rural	1	
Semi-urban/urban	2.91 (0.94–8.99)	0.064
**Age (years)**		
25–39	1	
40–49	0.81 (0.26–2.51)	0.711
**Education level**		
None/primary	1	
Post primary	0.57 (0.19–1.72)	0.316
**Occupation**		
Farming	1	
Others (Business, housewife, civil servant)	1.87 (0.68–5.17)	0.228
**Income**		
Below $40	1	
$40 and above	2.57 (0.83–7.97)	0.101
**Ever had an HIV test**		
No	1	
Yes	3.39 (0.41–27.89)	0.256
**Number of persons in household**		
Above five	1	
Five and below	1.43 (0.52–3.92)	0.480
**Place where reproductive health care was accessed**		
Private facility	1	
Government facility	5.21 (0.45–60.78)	0.188
**Ever been recommended for screening by health worker**		
No	1	
Yes	87.85 (30.28–254.84)	<0.001*
**Knew where cervical cancer screening was provided**		
No	1	
Yes	6.24 (1.81–21.56)	0.004*
**Knew that cervical cancer can be prevented**		
No	1	
Yes	2.72 (0.68–10.90)	0.156
**Knew more than one preventive measure for cervical cancer**		
No	1	
Yes	0.42 (0.13–1.35)	0.145
**Knew more than one symptom of cervical cancer**		
No	1	
Yes	0.51 (0.17–1.57)	0.243
**Knew atleast one test for cervical cancer**		
No	1	
Yes	2.04 (0.70–5.92)	0.243
**Knew someone who had ever been screened for cervical cancer**		
No	1	
Yes	9.48 (2.39–37.56)	0.001*
**Knew someone who had ever been diagnosed with cervical cancer**		
No	1	
Yes	0.55 (0.17–1.83)	0.332

* Statistically significant at p < 0.05.

### Increasing uptake of cervical cancer screening services

When respondents were asked about what could be done to increase uptake of cervical cancer screening services in their communities, majority 704 (78.2%) suggested increasing awareness about the disease, 399 (44.3%) said they should be provided with cervical cancer screening facilities while 143 (15.9%) requested for more female staff at the screening health facilities (Table 6).

**Table 6:** Suggested measures for increasing uptake of cervical cancer screening services.

Suggestions to increase uptake of screening services	Frequency (%)
Increase awareness about cervical cancer and screening	704 (78.2)
Provision of cervical cancer screening facilities	399 (44.3)
Cheaper cost for screening service	170 (18.9)
More female staff at screening facilities	143 (15.9)
Extend screening services nearer to communities	83 (9.2)

## Discussion

This study provides insights in to the level of cervical cancer screening and associated factors among women in rural Uganda. We found that only 4.8% of respondents had ever screened for cervical cancer. This low level of screening is similar with the 5-year screening prevalence for developing countries estimated by WHO (5%) [24] and in close agreement with a prevalence of 6% reported by a Kenyan [11] and Tanzanian study [25], and 7% by a Ugandan study [26]. Other studies in East Africa have reported higher proportions ranging from 12% to 27% [12, 14, 15, 18]. Many of the previous studies were conducted in health care settings except the Ugandan and Tanzanian studies.

The independent predictors for cervical cancer screening were: being recommended for screening by a health worker, knowing where cervical cancer screening services were offered and knowing someone who had ever been screened for the disease. Similar predictors for cervical cancer screening have been reported in previous studies. Indeed, studies carried out in Uganda [25], Jamaica [27, 28] and the United States [26] found that women who had been recommended for screening by a health worker were more likely to be screened. Other studies found an association between awareness of cervical cancer services and undergoing screening [25, 27–29]. Additionally, a multitude of studies have shown that women’s decisions to screen is influenced by experiences of their friends or peers [28, 30–32].

Although the proportion of women who had accessed screening was very low, knowledge about cervical cancer and its risk factors was high as reported in an earlier study [33]. Previous studies are a testament that knowledge may not necessarily result in to practice [15, 16, 18] as intermediary factors like attitudes may play an important role in formulating behaviour [34]. In fact, in our study, the major barriers to cervical cancer screening respondents reported were perception-related including having no signs and symptoms of the disease, thought of not being at risk, lack of time and fear of test outcomes. Similar barriers to cervical cancer screening have been reported elsewhere [12, 18, 21, 29]. Education campaigns should focus on improving such attitudes, increasing risk perceptions and encouraging women to seek screening even when free from signs and symptoms of the disease.

The finding that women who had been recommended for screening by a health worker were over eighty times more likely to have been screened for the disease is an indication that most women only got screened after they had been told to do so by a health worker. This presents both a challenge and an opportunity. A challenge that many times cervical cancer is diagnosed in its late stages as most women would not have accessed the service until it is late. The opportunity presented is that health workers can be used as an effective intervention to increase utilisation of screening services among women. Indeed, in this study, women reported that health workers were a significant source of the information they had about cervical cancer [33], similar to previous studies [11, 14, 18]. Moreover, higher intentions to screen have been recorded among women who reported discussions on cervical cancer with health care providers [26, 28, 29, 35]. Cervical cancer screening should therefore form part of the discussion between health workers and women when they go to seek health care. This could take the form of asking patients whether they have ever screened during routine visits, providing them with more information and support, and recommending them to access cervical cancer screening services.

In our study, respondents who knew a place where cervical cancer screening was offered were more likely to have been screened. This being a cross sectional study, it is hard to determine whether the respondents accessed screening because they knew where the service was offered or got to know such places because they had been screened themselves. Moreover, women who had not been screened pointed out the lack of awareness of the service as a significant barrier. The place of residence was also associated with cervical cancer screening as respondents who resided in urban or semi urban areas were more likely to have been screened. However, this association was confounded by occupation at multivariate analysis. Access to cervical cancer screening in rural areas has been shown to be more difficult due to health centres not being within walking distances, the lack of transport and cost of transportation [21, 25]. This study also found that women who accessed reproductive health care from government facilities were more likely to have been screened for cervical cancer when compared to those at private facilities at bivariate analysis. This could be due to the fact that cervical cancer screening service is free at the government health facilities that offer it unlike the private ones. This shows that if cervical cancer screening services are scaled up and the identified barriers addressed, more women would access screening. Also in the study, respondents who knew someone who had ever been screened were most likely to have been screened. This underscores the importance of social influence in promoting cervical cancer screening. Social influence is a process where people directly or indirectly influence thoughts, feelings and action of others [36]. Information about cervical cancer should also be targeted to social groups in the community such as women and youth groups to encourage increased utilisation of screening services.

This study had some limitations. First, being a cross sectional study, it is not possible to assess causality. Secondly, this study was carried out in two majorly rural districts and therefore the findings may not be generalizable to other contextually different areas. Thirdly, cervical cancer screening status was self-reported and could have been affected by social desirability. However, potential bias was minimised by asking respondents to provide dates when they accessed the service and duration since they last accessed it, which ensured reliability and validity of the data. Lastly, although the study had a large sample size (N = 900), there was low uptake of cervical cancer screening (4.8%), which affected the statistical tests and led to some wide confidence intervals.

## Conclusion

This study found a very low level of cervical cancer screening among women in rural Uganda. The barriers to screening identified included not being aware of cervical cancer screening services, health facility related challenges such as distance to health facilities and costs of the service, and individual perceptions related to; having no signs and symptoms of the disease, not being at risk, lack of time and fear of test outcomes. The independent predictors for cervical cancer screening were being recommended for screening by a health worker, knowing where cervical cancer screening services were offered and knowing someone who had ever been screened for the disease. There is need to increase access to cervical cancer screening in rural areas so as to increase utilisation of the service. In addition, cervical cancer screening can be increased by utilising health workers to discuss the disease with women when they go to seek health care.
